# Triamterene induces autophagic degradation of lysosome by exacerbating lysosomal integrity

**DOI:** 10.1007/s12272-021-01335-5

**Published:** 2021-06-07

**Authors:** Na Yeon Park, Doo Sin Jo, Yong Hwan Kim, Ji-Eun Bae, Joon Bum Kim, Hyun Jun Park, Ji Yeon Choi, Ha Jung Lee, Jeong Ho Chang, Heeyoun Bunch, Hong Bae Jeon, Yong-Keun Jung, Dong-Hyung Cho

**Affiliations:** 1 School of Life Sciences, BK21 FOUR KNU Creative BioResearch Group, Kyungpook National University, 80 Daehakro Bukgu, Daegu, 41566 Republic of Korea; 2 Brain Science and Engineering Institute, Kyungpook National University, Daegu, 41566 Republic of Korea; 3 Department of Biology Education, Kyungpook National University, Daegu, 41566 Republic of Korea; 4 School of Applied Biosciences, College of Agriculture and Life Science, Kyungpook National University, Daegu, 41566 Republic of Korea; 5 Stem Cell Institute, ENCell Co. Ltd, Seoul, 06072 Republic of Korea; 6 School of Biological Sciences, Seoul National University, Seoul, 08826 Republic of Korea

**Keywords:** Triamterene, Lysophagy, Lysosomal integrity, Autophagy, LLOMe, HepG2 cells

## Abstract

The maintenance of lysosomal integrity is essential for lysosome function and cell fate. Damaged lysosomes are degraded by lysosomal autophagy, lysophagy. The mechanism underlying lysophagy remains largely unknown; this study aimed to contribute to the understanding of this topic. A cell-based screening system was used to identify novel lysophagy modulators. Triamterene (6-phenylpteridine-2,4,7-triamine) was identified as one of the most potent lysophagy inducers from the screening process. We found that triamterene causes lysosomal rupture without affecting other cellular organelles and increases autophagy flux in HepG2 cells. Damaged lysosomes in triamterene-treated cells were removed by autophagy-mediated pathway, which was inhibited by depletion of the autophagy regulator, ATG5 or SQSTM1. In addition, treatment of triamterene decreased the integrity of lysosome and cell viability, which were rescued by removing the triamterene treatment in HepG2 cells. Hence, our data suggest that triamterene is a novel lysophagy inducer through the disruption of lysosomal integrity.

**Supplementary Information:**

The online version contains supplementary material available at 10.1007/s12272-021-01335-5.

## Introduction

Lysosomes, single membrane-bound organelles, are responsible for various cellular functions, including the degradation of extra- or intracellular materials, nutrient sensing and recycling, cholesterol homeostasis, and cell death (Settembre et al. 2013). As the main organelles of degradation, lysosomes contain more than 60 degradative enzymes, such as proteases, lipases, nucleases, and other hydrolases, which are optimally activated in acidic pH conditions (Perera and Zoncu 2016). According to this notion, disruption of the lysosome results in increased cytosolic acidity, and leakage of these enzymes into the cytoplasm can trigger cell death (Kroemer and Jäättelä 2005; Papadopoulos and Meyer 2017). Therefore, the maintenance of lysosomal function and integrity is essential for cellular homeostasis. Various lysosomotropic agents induce lysosomal membrane permeabilization (LMP) under conditions of stress, releasing lysosomal contents (Boya and Kroemer 2008), including monosodium urate, pathogens, lipids, β-amyloid, and membrane-disturbing agents, such as l-leucyl-l-leucine methyl ester (LLOMe) (Papadopoulos and Meyer 2017).

Macroautophagy (Autophagy) is a lysosome-dependent quality control mechanism that degrades cellular contents in response to various cellular stresses (Dikic and Elazar 2018; Pun et al. 2020). Autophagy begins with the isolation membrane, phagophore, gradually elongates to form a mature autophagosome (Mizushima and Komatsu 2011). After maturation, the autophagosome fuses with the lysosome to degrade molecules (Mizushima and Komatsu 2011; Dikic and Elazar 2018). The autophagic process is regulated by a series of autophagy-related (ATG) proteins (Dikic and Elazar 2018), and autophagy can selectively eliminate specific components, referred to as selective autophagy. For example, protein aggregates and parts of or entire organelles, such as mitochondria (mitophagy), peroxisome (pexophagy), ER (ER-phagy), and Golgi (Golgi-phagy), can be targeted for selective autophagy (Jo and Cho 2019; Kirkin 2020). Lysosomes are also degraded by selective autophagy, called lysophagy, in an ubiquitin-dependent manner (Hung et al. 2013; Maejima et al. 2013).

In response to lysosomal damage, such as membrane rupture, galectins and ubiquitin are recruited to lysosomes to initiate ‘eat-me’ signals, followed by lysophagy (Maejima et al. 2013). The lumen of a lysosome is maintained at a low pH for normal function; the membrane is protected from this environment through the generation of glycocalyx from the glycosylated proteins on the membrane (Perera and Zoncu 2016). Galectins have a high affinity for β-galactosides. Upon lysosomal membrane disruption, β-galactose-containing glycoconjugates in the lumen of lysosomes are exposed and further recognized by galectins (Thurston et al. 2012; Maejima et al. 2013; Aits et al. 2015). According to this notion, galectin-3 (Gal3) has been widely used as a monitoring marker for damaged lysosomes (Maejima et al. 2013; Aits et al. 2015; Papadopoulos et al. 2017).

Ubiquitination is also involved in the regulation of lysophagy. Damaged lysosomes labeled with Gal3 are poly-ubiquitinated (Maejima et al. 2013). The E3 ligases, tripartite motif 16 (TRIM16), and valosin containing protein (VCP) play a role in the ubiquitination of ruptured lysosomes (Bell et al. 2012; Chauhan et al. 2016; Papadopoulos et al. 2017). In addition, ubiquitin conjugating enzyme E2 Q family like 1 (UBE2QL1) also contributes to the ubiquitination of lysosomes in response to lysosomotropic agents. Loss of UBE2QL1 decreases the ubiquitination of lysosomes, formation of autophagosomes, and recruitment of VCP (Koerver et al. 2019). This ubiquitination is followed by the recruitment of autophagy receptors, such as SQSTM1/p62, which bind to ubiquitinated targets and the LC3 protein, leading to autophagic degradation (Anding and Baehrecke 2017). The SQSTM1 protein is recruited on ruptured lysosomes and functions as a receptor for lysophagy (Maejima et al. 2013; Papadopoulos et al. 2017).

Although several regulators involved in lysophagy have been identified, the molecular mechanisms underlying lysophagy and its inducers are still poorly understood. This study revealed that triamterene (6-phenylpteridine-2,4,7-triamine/C_12_H_11_N_7_) increased lysosomal damage and decreased lysosomal integrity in HepG2 cells. The inhibition of autophagy was also found to suppress the clearance of damaged lysosomes in triamterene-treated cells. These results indicated that triamterene is a novel lysophagy regulator participating in the molecular mechanism of lysophagy.

## Materials and methods

### Reagents

l-leucyl-l-leucine methyl ester (LLOMe), triamterene, amiloride hydrochloride hydrate, aminopterin, pemetrexed disodium heptahydrate, and bafilomycin A_1_ were purchased from Sigma-Aldrich (St. Louis, MO, USA). Torin1 was purchased from TOCRIS (Bristol, UK). SBI-115 was purchased from Biovision (Milpitas, CA, USA). Cell Counting Kit-8 (CCK-8) was purchased from Dojindo (Rockville, MD, USA). The short interfering RNA (siRNA) targeting for *ATG5* (5′-CAGGUAAGUCAAGCCUACAUU-3′), *SQSTM1* (5′-GCAUUGAAGUUGAUAUCGAUUU-3′), and negative scrambled siRNA (5′-CCUACGCCACCAAUUUCGU-3′) were synthesized by Genolution (Seoul, Korea).

### Plasmids

The expression plasmids pEGFP-hGalectin-3 (GFP-Gal3) and pmRFP-EGFP-Galectin-3 (ptf-Gal3) were purchased from Addgene [deposited by Dr. Tamotsu Yoshimori (Osaka University, Japan)]. The subcellular localization vectors, pmTurquoise2-ER, pmTurquoise2-Golgi, and pmTurquoise2-Peroxi, in which a cyan fluorescence protein (Turquoise) was fused with ER, Golgi, or peroxisome targeting sequences respectively, were obtained from Addgene [deposited by Dr. Dorus Gadella (University of Amsterdam, Netherlands)]. pEGFP-TFEB was obtained from Addgene [deposited by Dr. Shawn Ferguson (Yale Univeristy, USA)]. pLAMP1-GFP and pEGFP-LC3 were kindly provided by Dr. Peter K. Kim (Toronto University, Canada) and Dr. Noboru Mizhushima (Tokyo University, Japan), respectively. pCMV-lyso-pHluorin (Lyso-pHluorin) was purchased from Addgene [deposited by Dr. Christian Rosenmund (Neuroscience Research Center, Germany)]. pEGFP-Ubiquitin (GFP-Ub) and pLAMP1-mCherry were purchased from Addgene [deposited by Dr. Nico Dantuma (Karolinska Institutet, Sweden) and Amy Pamer (University of Colorado, USA)], respectively. Mitochondrial-YFP (pMito-YFP) plasmid was kindly provided by Dr. Gyesoon Yoon (Ajou University, Korea).

### Cell culture and establishment of stable cell lines

HepG2 and HeLa cells were obtained from the American Type Culture Collection. The ATG5-deficient HeLa cells generated with the CRISPR/Cas9 system (HeLa/ATG5 KO) were kindly provided by Dr. Tomotake Kanki (Niigata University, Japan). To generate stable cell lines, HepG2 cells were transfected with either GFP-Gal3 or ptf-Gal3 using Lipofectamine 2000 (Invitrogen, Carlsbad, CA, USA) according to the manufacturer’s protocol. Stable transfectants were selected using 1 mg/ml of G418 for 10 days (Invitrogen). After seeding the individual cells, the stable clones were selected under a fluorescence microscope (Olympus, Tokyo, Japan).

### Cell-based fecal metabolites library screening

For the cell-based fecal metabolites library screening, HepG2/GFP-Gal3 cells were seeded in a 96-well plate. After 24 h, approximately 100–500 µM of MetaSci Fecal Metabolites Library (MetaSci, Toronto, Canada), including endogenous metabolites and various bioactive chemicals, was added to each well. GFP-Gal3 puncta in the cells were monitored by fluorescence microscopy (Olympus). The experiments were repeated 3 times at the same condition.

### Western blotting

For immunoblotting, cells were harvested using a cell lysis buffer and prepared with 2× Laemmli sample buffer (Bio-Rad, Hercules, CA, USA). Total protein quantity was measured using Bradford solution (Bio-Rad) according to the manufacturer’s instructions. Then, the samples were separated by SDS-PAGE and transferred to a PVDF membrane (Bio-Rad). After blocking with a 4% skim milk (MBcell, Seoul, Korea) in TBST [25 mM Tris-base (GenDEPOT, Katy, TX, USA), 140 mM NaCl (GenDEPOT), and 0.05% Tween® 20 (Sigma)], the membrane was probed with the following primary antibodies: anti-LC3 and anti-ATG5 antibodies, purchased from NOVUS Biologicals (Centennial, CO, USA); anti-SQSTM1 and anti-p-TFEB antibodies, purchased from Cell Signaling Technology (Danvers, MA, USA); anti-GFP, anti-LAMP1, anti-P4HB, and anti-TOMM20 antibodies purchased from Santa Cruz (Dallas, TX, USA); anti-FTCD, and anti-ABCD3 antibodies purchased from Abcam (Cambridge, UK); anti-α-Actin (ACTA1) antibody, purchased from Sigma. For protein detection, the membranes were incubated with HRP-conjugated secondary antibodies (Cell Signaling Technology).

### Fluorescence microscopy

Cells were treated with triamterene and fixed with 4 % paraformaldehyde for 10 min. Samples were then observed using a fluorescence microscope (Olympus). The number of Gal3 puncta was analyzed by Image J (NIH, Bethesda, MD, USA). Analysis of graph data was performed with GraphPad Prism 8 (GraphPad Software, San Diego, CA, USA).

### Lysosomal acidification measurement

HepG2 cells were transiently transfected with lyso-pHluorin using Lipofectamine 2000 (Invitrogen) according to the manufacturer’s protocol. Next, the cells were treated with LLOMe or triamterene for 2 h. After 2 h, triamterene was washed out and changed with normal cell culture media and incubated for another 6 and 24 h. The fluorescent signals resulting from each chemical were captured using a fluorescence microscope (Olympus). The intensity of fluorescence was measured by Image J, and the analysis of graph data was performed with GraphPad Prism 8.

### Cell viability assay

HepG2 cells cultured in 96-well plates were incubated with either LLOMe (750 µM) or 200 µM triamterene for 2 h then washed with fresh medium and incubated for 6 and 24 h. In addition, HeLa and HeLa/ATG5 KO cells were cultured in 96-well plates and incubated with either LLOMe or triamterene for 12 h. Using a CCK-8 following the manufacturer’s protocol, the cell viability was calculated as follows: (chemical treated cells)/(untreated cells) × 100.

### Statistical analysis

Data were obtained from at least 3 independent experiments, and presented as means ± S.E.M. Statistical evaluation of the results was performed using one-way ANOVA. Data were considered significant at with *p* values of *p < 0.05, **p < 0.01, and ***p < 0.001.

## Results

### Triamterene increases lysosomal rupture

**Fig. 1:**
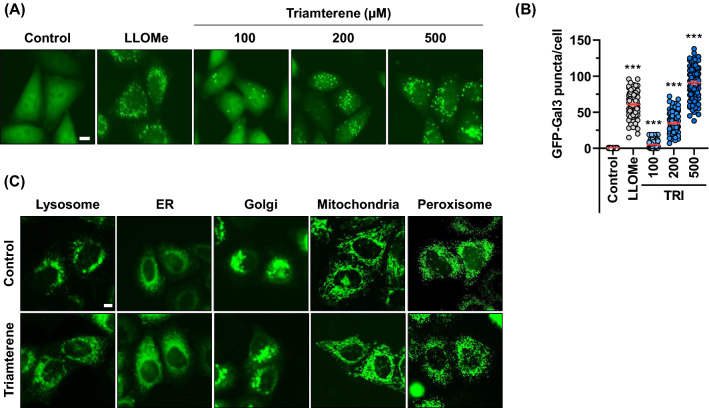
Triamterene induces GFP-Galectin3 puncta in HepG2 cells. **A**, **B** HepG2 cells stably expressing GFP-Gal3 (HepG2/GFP-Gal3) were treated with either LLOMe (750 µM) or 100, 200, 500 µM of triamterene (TRI) for 2 h. **A** Cells were imaged by fluorescence microscopy, **B** and the number of Gal3 puncta per cell were counted (n = 100, Data are presented as the mean ± SEM (***p < 0.001). **C** Transiently transfected HepG2 cells with markers for cellular organelles, LAMP1-GFP (Lysosome), turquoise2-ER (ER), turquoise2-Golgi (Golgi), Mito-YFP (Mitochondria), and turquoise2-Peroxi (Peroxisome) were treated with triamterene (200 µM) for 2 h or not and imaged by fluorescence microscopy. Scale bar: 10 μm

Although lysophagy has emerged as a dedicated response to changes in permeability and the integrity of lysosomes, the precise regulatory mechanism is mostly unknown. To identify new regulators of lysophagy, GFP-tagging hGalectin-3 (GFP-Gal3) has been used as a lysosomal damage monitoring system. Because Gal3 is widely used to monitor lysosome damage (Paz et al. 2010; Maejima et al. 2013), we generated a stable HepG2 cell line expressing GFP-Gal3 (HepG2/GFP-Gal3) and screened a library containing ~ 550 metabolites and chemical drugs. From the screening, we identified triamterene as well as several putative regulators for lysophagy (Supplementary Fig. 1). To confirm the screening results, HepG2/GFP-Gal3 cells were treated with triamterene at different concentrations (100–500 µM) (Fig. 1 A). LLOMe (750 µM) was used as a positive control to disrupt the lysosomal membrane (Maejima et al. 2013). Treatment had no influence to increase of Gal3-puncta in lower concentration, while 100–200 µM triamterene efficiently induced Gal3-puncta formation (Fig. 1A and B). As higher dosage of triamterene (> 500 µM) showed cytotoxicity in HepG2 cells, we used 100–200 µM of triamterene in further studies (Data not shown). The number of Gal3 puncta per cell was also significantly increased in triamterene-treated cells in a dose-dependent manner (Fig. 1B). To further investigate whether triamterene treatment influences other organelles, we observed cellular organelles, including mitochondria, ER, Golgi apparatus, and peroxisome in triamterene-treated cells. Cells expressing organelle marker proteins, such as LAMP1-GFP (lysosome), turquoise2-ER (ER), turquoise2-Golgi (Golgi), mito-YFP (mitochondria), and turquoise2-Peroxi (peroxisome), were treated with triamterene and monitored. The results showed that ER, Golgi, mitochondria, and peroxisome were not notably changed by treatment with triamterene in HepG2 cells, while some swelling was observed in lysosomes (Fig. 1 C).

### Triamterene decreases integrity of lysosomes

**Fig. 2:**
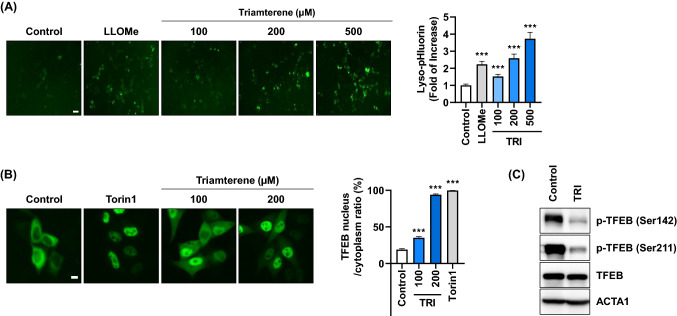
Triamterene decreases integrity of lysosome but increases translocation of TFEB in HepG2 cells. **A** HepG2 cells expressing Lyso-pHluorin were treated with triamterene (TRI, 100, 200, 500 µM) for 2 h. The fluorescence intensity of pHluorin protein in the cells was imaged and counted. Data are presented as the mean ± SEM (n = 20, ***p < 0.001). Scale bar: 10 μm. **B** Transiently transfected HepG2 cells with TFEB-GFP were treated with triamterene (TRI, 100, 200 µM) or Torin1 (1 µM) for 2 h. Localization was imaged by fluorescence microscope, and the ratio (nucleus/cytosol, n = 10) was counted. Data are presented as the mean ± SEM (***p < 0.001). Scale bar: 10 μm. **C** The cells were harvested and phosphorylation status of TFEB was analyzed by western blotting

As a degradative organelle, lysosomal integrity is important for cell homeostasis and function (Papadopoulos and Meyer 2017). To investigate the lysosomal pH level in triamterene-treated cells, we employed a lyso-pHluorin system (Rost et al. 2015). The pHluorin emits a green fluorescent signal at neutral pH; it is easily quenched under the acidic conditions within the lysosomal lumen (Miesenböck et al. 1998). Cells expressing lyso-pHluorin were treated with triamterene or LLOMe, and the fluorescent intensity was monitored. As shown in Fig. 2A, the fluorescent signal of pHluorin dose-dependently increased in triamterene-treated cells. The transcription factor EB (TFEB) coordinates lysophagy by driving the expression of autophagy and lysosomal genes (Settembre et al. 2011). Thus, in response to autophagy activation, TFEB is dephosphorylated, which promotes its nucleus translocation to activate target genes (Napolitano and Ballabio 2016). As TFEB is activated to recover lysosomal damage (Jia et al. 2018; Zhitomirsky et al. 2018), we further examined the TFEB activation in triamterene-treated cells. Similar to Torin1, a potent mTOR inhibitor, treatment with triamterene efficiently induced the translocation of TFEB to the nucleus from the cytosol (Fig. 2B). Furthermore, we found that treatment with triamterene induces dephosphorylation of TFEB (Fig. 2C), suggesting triamterene activates TFEB in HepG2 cells.

### Triamterene activates lysophagy in HepG2 cells

**Fig. 3:**
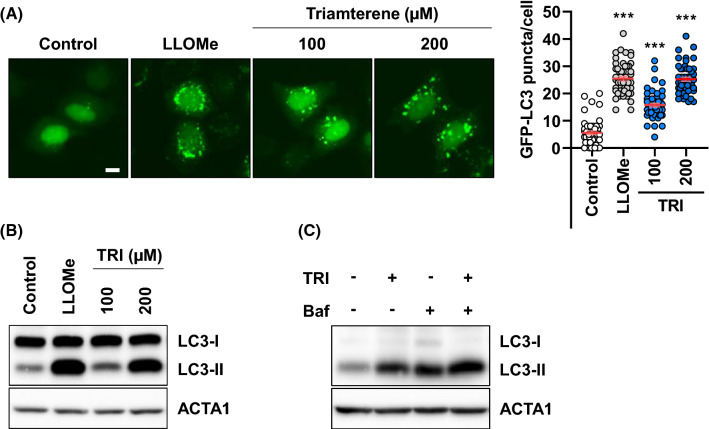
Triamterene enhances autophagic flux in HepG2 cells. **A**, **B** HepG2 cells transiently expressing GFP-LC3 were treated with triamterene (TRI, 100, 200 µM). **A** The cells were imaged by fluorescence microscopy, and the number of LC3 puncta per cell was counted. **B** The cells were analyzed by western blotting with anti-LC3 antibody and LLOMe (750 µM) (positive control). **C** Triamterene (TRI)-treated HepG2 cells incubated in the presence or absence of bafilomycin A_1_ (Baf) were analyzed by western blotting for LC3 conversion. Data are presented as the mean ± SEM (n = 50, ***p < 0.001). Scale bar: 10 μm

**Fig. 4:**
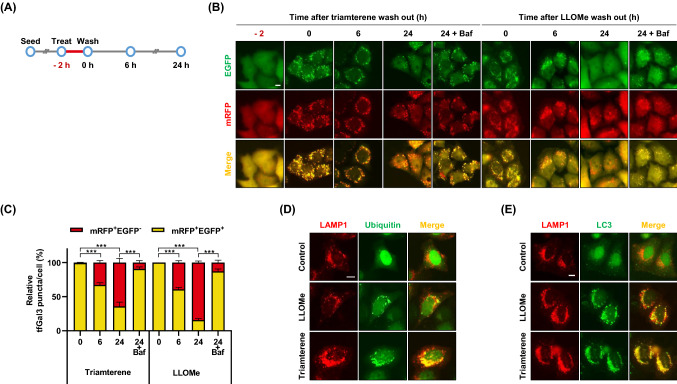
Triamterene induces lysophagy. **A** Schematic diagram of the experimental design. **B** HepG2 cells stably expressing tandem fluorescence-tagged Galectin-3 (ptf-Gal3) (HepG2/ptf-Gal3) were treated with either LLOMe (750 µM) or triamterene (200 µM). After 2 h, the chemicals were washed out, and the cells were incubated with normal cell culture media in the presence or absence of bafilomycin A_1_ (Baf, 30 nM). And then, the cells were imaged at the indicated time points by a fluorescence microscope. **C** Cells with mRFP- and EGFP-double-positive-Gal3 puncta (mRFP^+^/EGFP^+^) or mRFP positive/EGFP negative-Gal3 puncta (mRFP^+^/EGFP^−^) were counted. Data are presented as the mean ± SEM (n = 10, *** *p* < 0.001). Scale bar: 10 μm. **D**, **E** Transiently cotransfected HepG2 cells with pLAMP1-mCherry and pEGFP-Ubiquitin (**D**) or pEGFP-LC3 (**E**) for 24 h were treated with either LLOMe (750 µM) or triamterene (200 µM) for 2 h. The cells were imaged by fluorescence microscopy. Scale bar: 10 μm

We next examined the effect of triamterene in autophagy activation. HepG2 cells expressing GFP-LC3, a marker for autophagy activation, were treated with either triamterene or LLOMe. As shown in Fig. 3A and B, both the formation of punctate GFP-LC3 protein and the conversion of LC3-II from LC3-I were dramatically increased in triamterene-treated and LLOMe-treated cells (Fig. 3A, B). To examine autophagy flux by triamterene treatment, HepG2 cells were treated with or without bafilomycin A_1_ (Baf), an inhibitor of autophagosome fusion to the lysosome. We found that the protein level of LC3-II was higher in cells treated with triamterene and bafilomycin A1 than cells treated with either of these compounds alone (Fig. 3C). Since triamterene appears to highly affect the lysosome rather than other organelles (Fig. 1C), we next investigated the effect of triamterene on lysophagy. To examine lysophagy induction, we developed a lysophagy monitoring system with mRFP- and GFP-tandem-tagged Gal3 (ptf-Gal3) in HepG2 cells (HepG2/ptf-Gal3). The outcome of this assay is based on the differences in pH sensitivity between the green- and red fluorescent proteins. The EGFP signal is easily quenched, while mRFP is more stable in lysosomal acidic conditions (Zhou et al. 2012; Maejima et al. 2013). Using this system, we found that most of the cells showed EGFP- and mRFP-double-positive-Gal3 puncta with either LLOMe or triamterene treatment (Fig. 4A–C). However, after removal of the chemicals, the number of cells with double-positive-Gal3 puncta decreased while mRFP-single-positive-Gal3 puncta cells increased, in a time-dependent manner (Fig. 4B, C). Moreover, the reduction of EGFP- and mRFP-double-positive-Gal3 puncta was efficiently blocked after treatment with Baf (Fig. 4B, C). It has been demonstrated that ubiquitination mediates selective autophagy for cellular organelles (Kirkin et al. 2009; Shaid et al. 2013). During selective autophagy, the ubiquitinated organelles are recognized by autophagy adaptor proteins, such as SQSTM1/p62 (Shaid et al. 2013). Throughout the literature, ubiquitin is recruited on damaged lysosomes to carry out lysophagy (Maejima et al. 2013; Papadopoulos et al. 2020). For these reasons, we investigated the role of ubiquitin in triamterene-induced lysophagy. As shown in Fig. 4D, ubiquitin was translocated and mainly colocalized with lysosomal protein, LAMP1, in triamterene-treated cells in contrast to control cells (Fig. 4D). In addition, LC3-labeled autophagosome also colocalized with lysosomes in LLOMe- or triamterene-treated cells (Fig. 4E). Taken together, these results suggest that triamterene enhances lysophagy in HepG2 cells.

### Depletion of ATG5 or SQSTM1 delays clearance of damaged lysosome in triamterene-treated cells

**Fig. 5:**
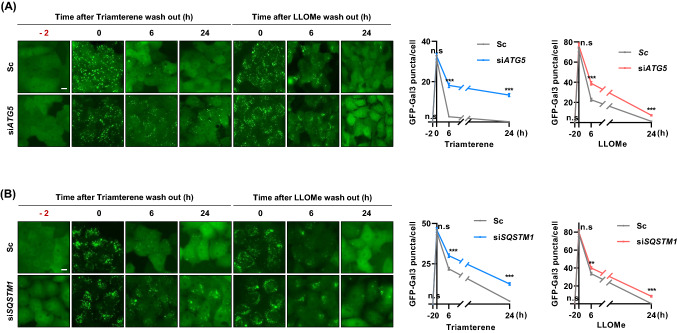
Triamterene-induced lysophagy is suppressed by depletion of ATG5 or SQSTM1. **A** HepG2/GFP-Gal3 cells were transfected with scrambled siRNA (Sc) or *ATG5*-targeting siRNA (si*ATG5*) for 72 h. Then, the cells were treated with either LLOMe (750 µM) or triamterene (200 µM) for 2 h. After washout of LLOMe and triamterene, the Gal3-puncta was observed with a fluorescence microscope at the indicated time points. Data are presented as the mean ± SEM (n = 100, ***p < 0.001). Scale bar: 10 μm. **B** HepG2/GFP-Gal3 cells were transfected with scrambled siRNA (Sc) or *SQSTM1*-targeting siRNA (si*SQSTM1*) for 72 h. The cells were then treated with either LLOMe (750 µM) or triamterene 200 µM for 2 h. After triamterene washout, cells were imaged and counted under a fluorescence microscope at the indicated time points. Data are presented as the mean ± SEM (n = 100, **p < 0.01, ***p < 0.001). Scale bar: 10 μm

Next, we investigated the effects of inhibition of autophagy on triamterene-induced lysophagy. HepG2/GFP-Gal3 cells transfected with scrambled siRNA or *ATG5* siRNA were pretreated with triamterene for 2 h, after which the cells were washed and incubated in normal culture media (Fig. 5A). As shown in Fig. 5A, the punctate structure of GFP-Gal3 by triamterene was dramatically cleared in the control cells in a time-dependent manner after washing. The depletion of ATG5 notably delayed the clearance of damaged lysosomes in triamterene-treated HepG2/GFP-Gal3 cells (Fig. 5A and Supplementary Fig. 2). LLOMe was used as a control. SQSTM1 plays a major role and is consistently found on damaged lysosomes (Maejima et al. 2013), and the downregulation of SQSTM1 impairs the clearance of damaged lysosomes (Papadopoulos et al. 2017). Thus, we further investigated the effect of triamterene in SQSTM1-depleted cells. Consistent with the ATG5 knockdown results, depletion of SQSTM1 significantly attenuates the clearance of damaged lysosomes in triamterene-treated cells (Fig. 5B and Supplementary Fig. 2). Taken together, these results suggested that triamterene induces lysophagy via the ATG5-and SQSTM1-dependent canonical autophagy pathway.

### Inhibition of lysophagy exacerbates cell death in triamterene-treated HepG2 cells

**Fig. 6:**
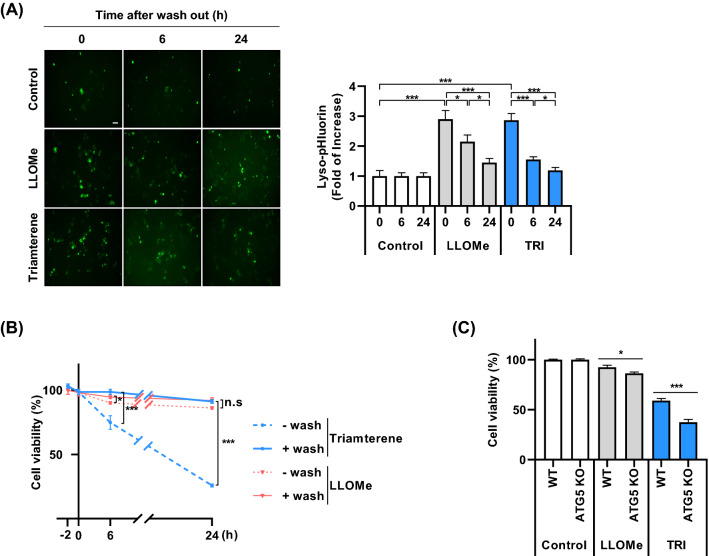
Inhibition of lysophagy potentiates cell death in triamterene-treated cells. **A** HepG2 cells expressing lyso-pHluorin were treated with either LLOMe (750 µM) or triamterene (TRI, 200 µM). After 2 h, LLOMe and triamterene were washed out, and the cells were incubated in normal cell culture conditions. The fluorescent of pHluorin in cells was imaged and measured at the indicated time points. Data are presented as the mean ± SEM (n = 20, *p < 0.05, ***p < 0.001). **B** HepG2 cells were treated with either LLOMe (750 µM) or triamterene 200 µM for 2 h. After 2 h, LLOMe and triamterene were washed out, and the cells were maintained with the media in the presence or absence of triamterene ([−] without wash and [+] with wash, respectively). The cell viability was measured by the CCK-8 assay. Data are presented as the mean ± SEM (n = 5, *p < 0.05, ***p < 0.001). **C** HeLa WT (WT) and HeLa/ATG5 KO (ATG5 KO) cells were treated with either LLOMe (750 µM) or triamterene (200 µM) for 12 h. Cell viability was measured by the CCK-8 assay. Data are presented as the mean ± SEM (n = 4, *p < 0.05, ***p < 0.001)

Because the integrity of lysosome is important for lysosome function, loss of lysosomal integrity affects cell viability (Papadopoulos and Meyer 2017; Arhzaouy et al. 2019); we investigated the effect of triamterene on lysosome integrity. HepG2 cells expressing lyso-pHluorin were treated with either LLOMe or triamterene. After 2 h, LLOMe and triamterene was replaced, and the cells were incubated in normal culture conditions for 6 and 24 h. Then, the fluorescent intensity of pHluorin was measured. The results showed that treatment with triamterene strongly reduced lysosomal integrity (Fig. 6A). The removal of triamterene significantly recovered lysosome integrity in a time-dependent manner (Fig. 6A). In addition, we examined whether the suppression of lysophagy affects cell death. Triamterene induced disruption of lysosomal integrity but decreased cell viability (Fig. 6B). However, the increase in cell death was completely blocked after triamterene was removed from the cells (Fig. 6B). To further investigate the effect of lysophagy in triamterene-mediated cell death, wild type and ATG5 knockout HeLa cells were treated with either LLOMe or triamterene, and cell viability was examined. Notably, we found that inhibition of lysophagy by depletion of ATG5 significantly exacerbated cell death in triamterene-treated cells compared to wild type control cells (Fig. 6C). However, other organelles were not significantly influenced by triamterene by in ATG5-deficient cells compared with that of wild type cells (Supplementary Fig. 3). Taken together, these results suggest that lysophagy contributes to cell survival after lysosome damage.

## Discussion

Lysosomes play key roles in various cellular functions including signaling, metabolism, and quality control of cellular components (Lawrence and Zoncu 2019). Various lysosomotropic agents promote the enlargement of the lysosome and ultimate LMP, which triggers cellular stress responses (Wang et al. 2018a, b). Although lysophagy improves cell survival by eliminating impaired lysosomes, there is little known about the underlying mechanisms. In this study, we identified triamterene as a strong lysophagy inducer using a cell-based screening method. We found that triamterene triggers lysosomal rupture and lysophagy in HepG2 cells (Figs. 1 and 3) and other types of cells, including HKC-8, HEK293, HCT116, RKO, and HeLa cells (data not shown). Blockage of autophagy by depletion of ATG5 suppressed the clearance of damaged lysosomes in triamterene-treated cells (Fig. 5). These findings suggest that triamterene induces autophagic degradation of lysosomes with LMP.

As cellular degradative organelles, the lumen of the lysosome maintains acidic conditions, achieved by a vascular ATPase (v-ATPase) in cooperation with ion channels (Zhao et al. 2015). Lysosomal acidification, essential for activation of lysosomal enzymes, is disrupted by LMP (Jessop et al. 2017). LMP results in the exposure of lysosomal contents, such as cathepsins, to the cytosol and triggers caspase-dependent cell death (Wang et al. 2018a). Thus, various lysosomotropic reagents such as LLOMe induce cell death by activating caspase (Wang et al. 2018a; Eriksson et al. 2020). It was previously reported that the loss of lysophagy mediating E3 ligases and E2 enzymes, such as VCP, TRIM16, and UBE2QL1, increased cytotoxicity (Chauhan et al. 2016; Papadopoulos et al. 2017; Koerver et al. 2019). Consistent with this notion, we also found that the inhibition of lysophagy exacerbated cell death in triamterene-treated cells (Fig. 6). As the role of lysophagy in cell survival is largely unknown, further studies on the triamterene-mediated lysophagy will provide insight into the role of lysophagy in cell death and survival.

Triamterene has been prescribed to patients with hypertension (Smetana 2016). It is a pteridine derivative and functions as a diuretic agent, where it decreases the excretion of potassium ions by inhibiting the epithelial sodium channel (ENaC) on the luminal side of the collecting tubule in the kidney (Smetana 2016). In addition, treatment with triamterene and its metabolites leads to folate deficiency by inhibiting dihydrofolate reductase in human leucocytes (Schalhorn et al. 1981). Therefore, triamterene induces the cytotoxicity in DNA mismatch repair (MMR) deficient tumor cells selectively by increasing reactive oxygen species through its antifolate activity (Guillotin et al. 2017). To investigate the effect of triamterene as a diuretic agent or antifolate molecule, we addressed lysophagy with some specific inhibitors for ENaC and folate reductase, such as amiloride, aminopterin, and pemetrexed (Anderson and Wright 2014; Sun and Sever 2020). However, we could not find any lysophagy inducing activity with inhibitors for ENaC and folate reductase (Supplementary Fig. 4). In addition to these activities, it was recently reported that triamterene has an antagonistic effect on G-protein-coupled bile acid receptor 1 (TGR5). Because glucose uptake induced by TGR5 agonists is inhibited by triamterene treatment (Li et al. 2017), we further examined the effect of TGR5 in lysophagy. However, treatment with SBI-115, a specific TGR5 antagonist (Masyuk et al. 2017), did not affect lysophagy in HepG2 cells (Supplementary Fig. 4). Though triamterene has been used to treat edema and hypertension (Smetana 2016), it causes nephrotoxin and acute kidney injury with several side effects, such as crystalluria, hyperuricemia, hyperkalemia, and even renal calculi (Nasr et al. 2014; Smetana 2016; Aghsaeifard and Alizadeh 2020). We hypothesized that triamterene contributes to lysosomal rupture and lysophagy through one specific action. Therefore, it is necessary to be cautious for clinical use of triamterene for hypertension and edema patients. Further investigation into the mechanism of signaling as well as its side effect for triamterene is needed to characterize and elucidate the underlying mechanisms regarding lysosome integrity and lysophagy. In conclusion, we found that triamterene leads to lysophagy by disruption of lysosomal integrity.

## Supplementary Information

Below is the link to the electronic supplementary material.

Electronic supplementary material 1 (PPTX 6112 kb)

Electronic supplementary material 2 (DOCX 13 kb)
