# Supplementary material for: Triamterene induces autophagic degradation of lysosome by exacerbating lysosomal integrity
Source: Arch Pharm Res. 2021 Jun 7;44(6):621–31. doi: 10.1007/s12272-021-01335-5 (PMC8254722; doi:10.1007/s12272-021-01335-5)
Supplement: Supplementary file 1 — Electronic supplementary material 1 (PPTX 6112 kb) [file 12272_2021_1335_MOESM1_ESM.pptx]

## Slide 1
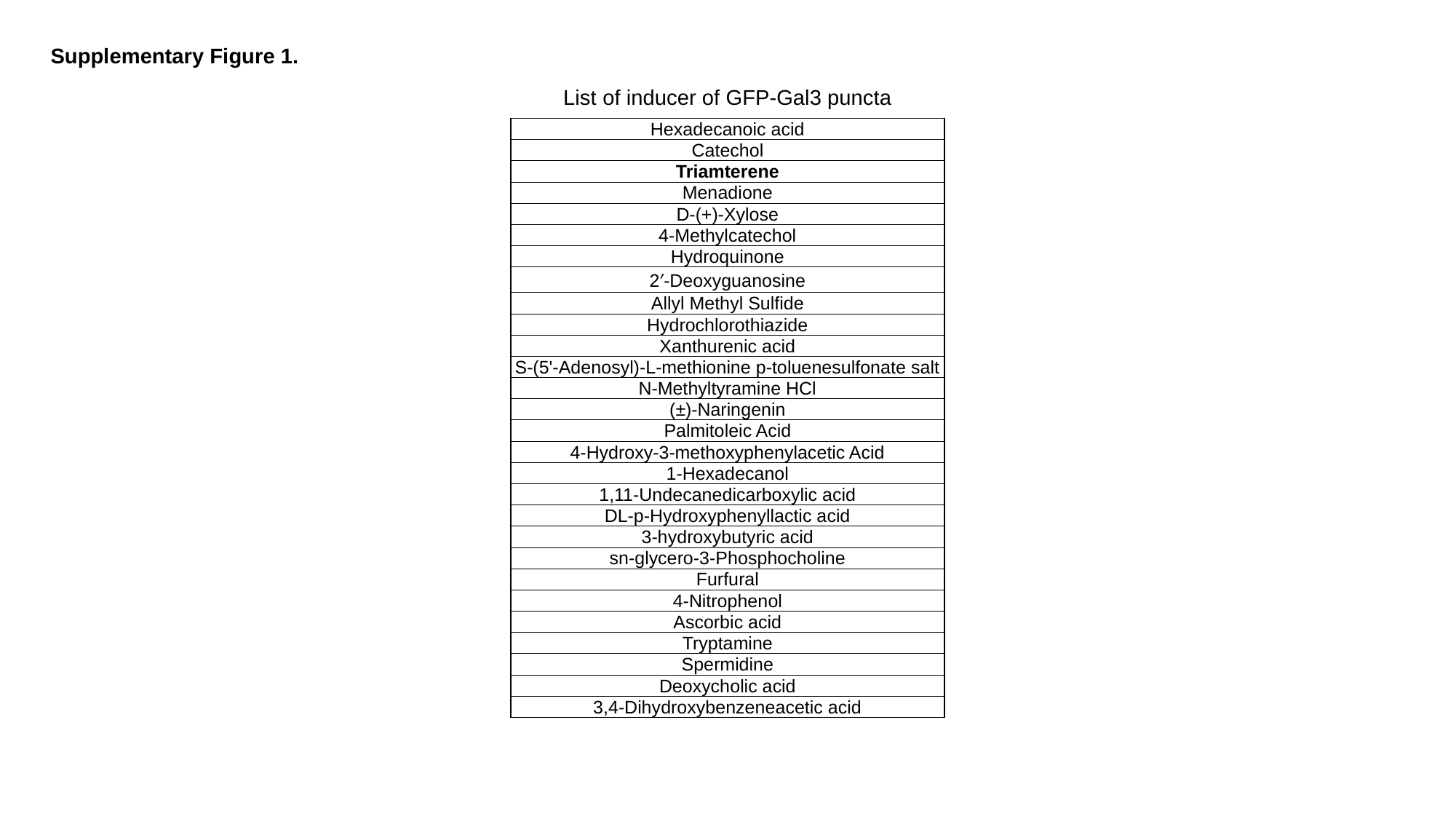

Supplementary Figure 1.
List of inducer of GFP-Gal3 puncta
| Hexadecanoic acid |
| --- |
| Catechol |
| Triamterene |
| Menadione |
| D-(+)-Xylose |
| 4-Methylcatechol |
| Hydroquinone |
| 2′-Deoxyguanosine |
| Allyl Methyl Sulfide |
| Hydrochlorothiazide |
| Xanthurenic acid |
| S-(5'-Adenosyl)-L-methionine p-toluenesulfonate salt |
| N-Methyltyramine HCl |
| (±)-Naringenin |
| Palmitoleic Acid |
| 4-Hydroxy-3-methoxyphenylacetic Acid |
| 1-Hexadecanol |
| 1,11-Undecanedicarboxylic acid |
| DL-p-Hydroxyphenyllactic acid |
| 3-hydroxybutyric acid |
| sn-glycero-3-Phosphocholine |
| Furfural |
| 4-Nitrophenol |
| Ascorbic acid |
| Tryptamine |
| Spermidine |
| Deoxycholic acid |
| 3,4-Dihydroxybenzeneacetic acid |

## Slide 2
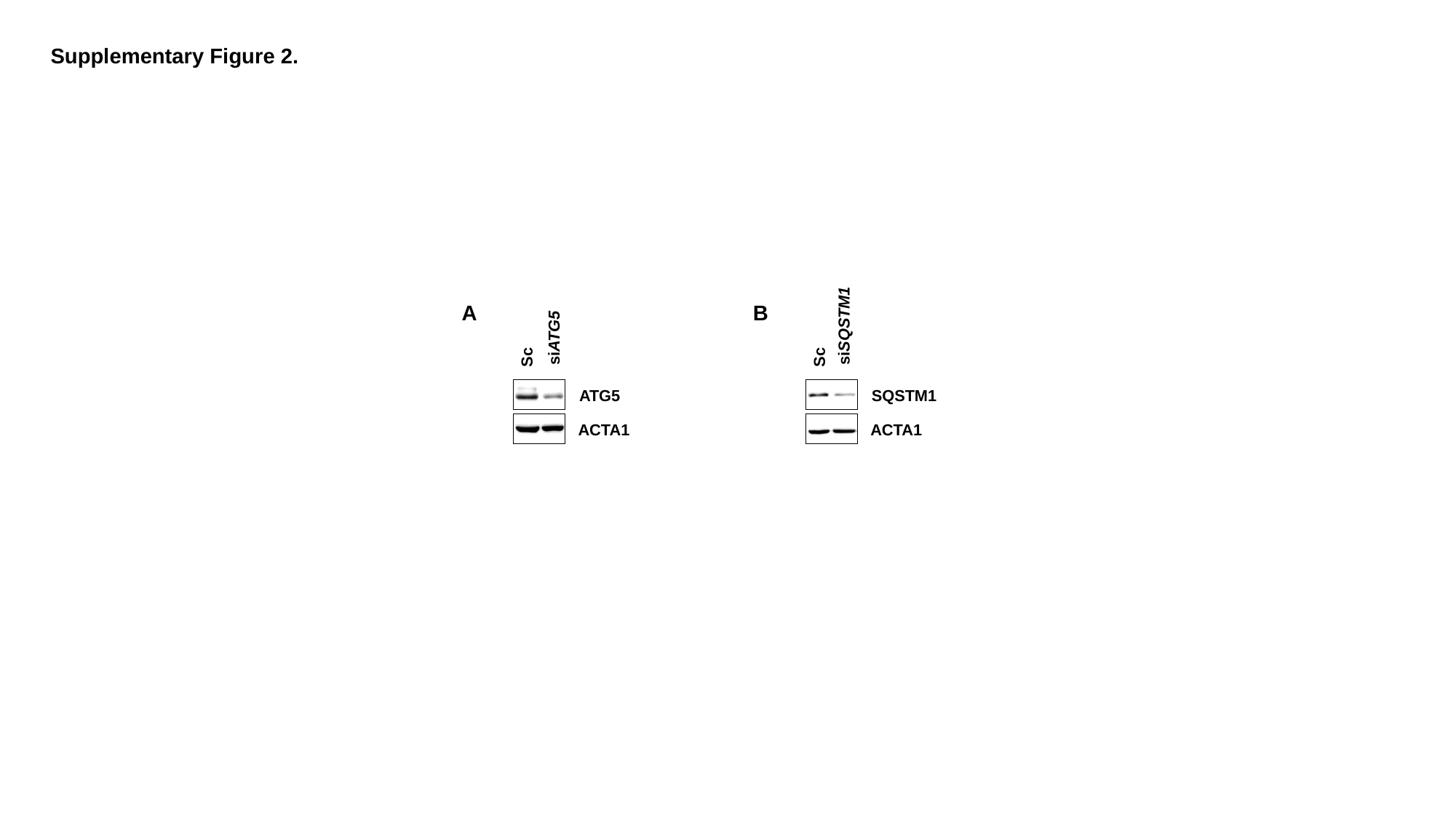

Supplementary Figure 2.
A
B
siSQSTM1
siATG5
Sc
Sc
ATG5
SQSTM1
ACTA1
ACTA1

## Slide 3
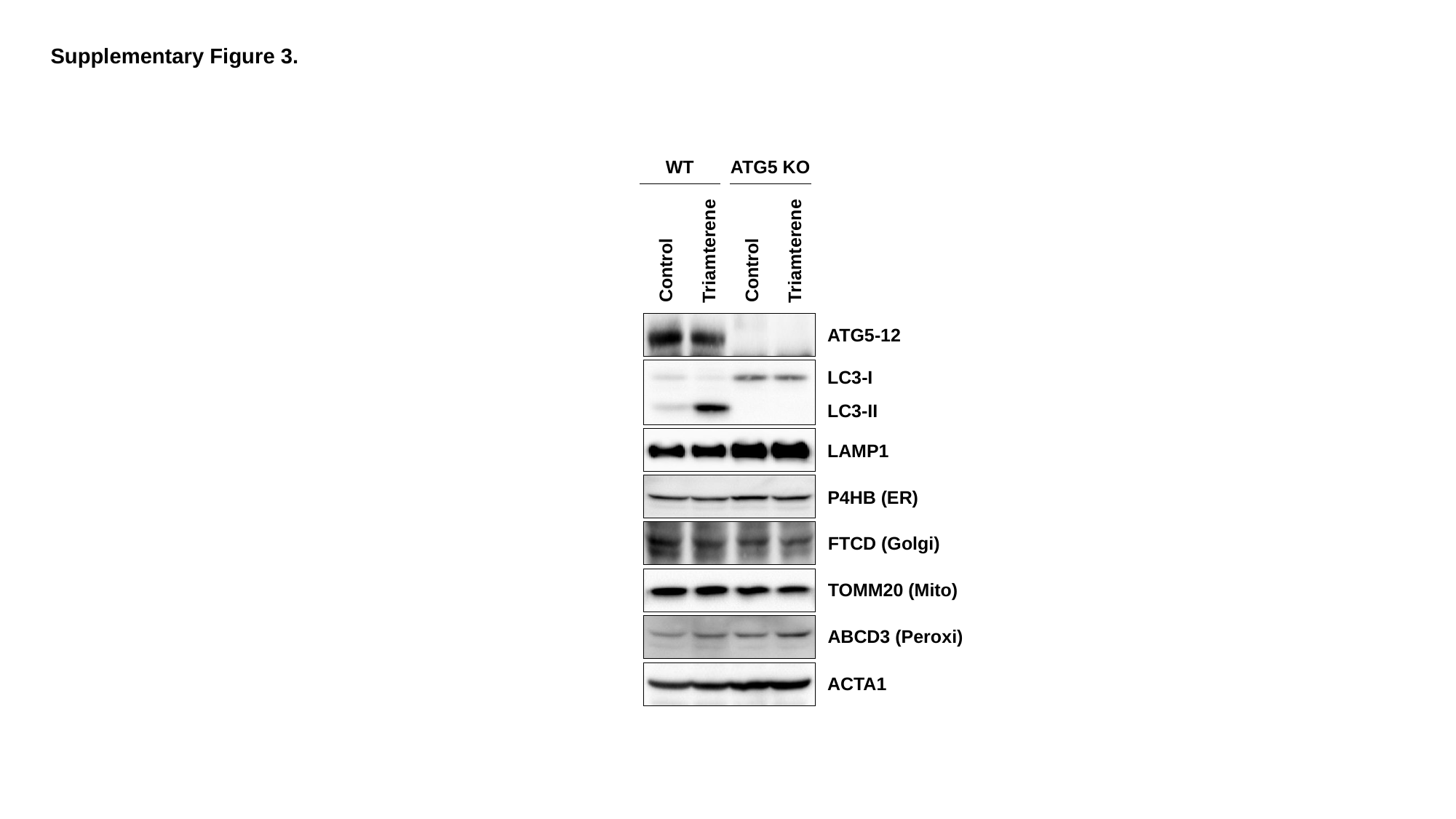

Supplementary Figure 3.
WT
ATG5 KO
Triamterene
Triamterene
Control
Control
ATG5-12
LC3-I
LC3-II
LAMP1
P4HB (ER)
FTCD (Golgi)
TOMM20 (Mito)
ABCD3 (Peroxi)
ACTA1

## Slide 4
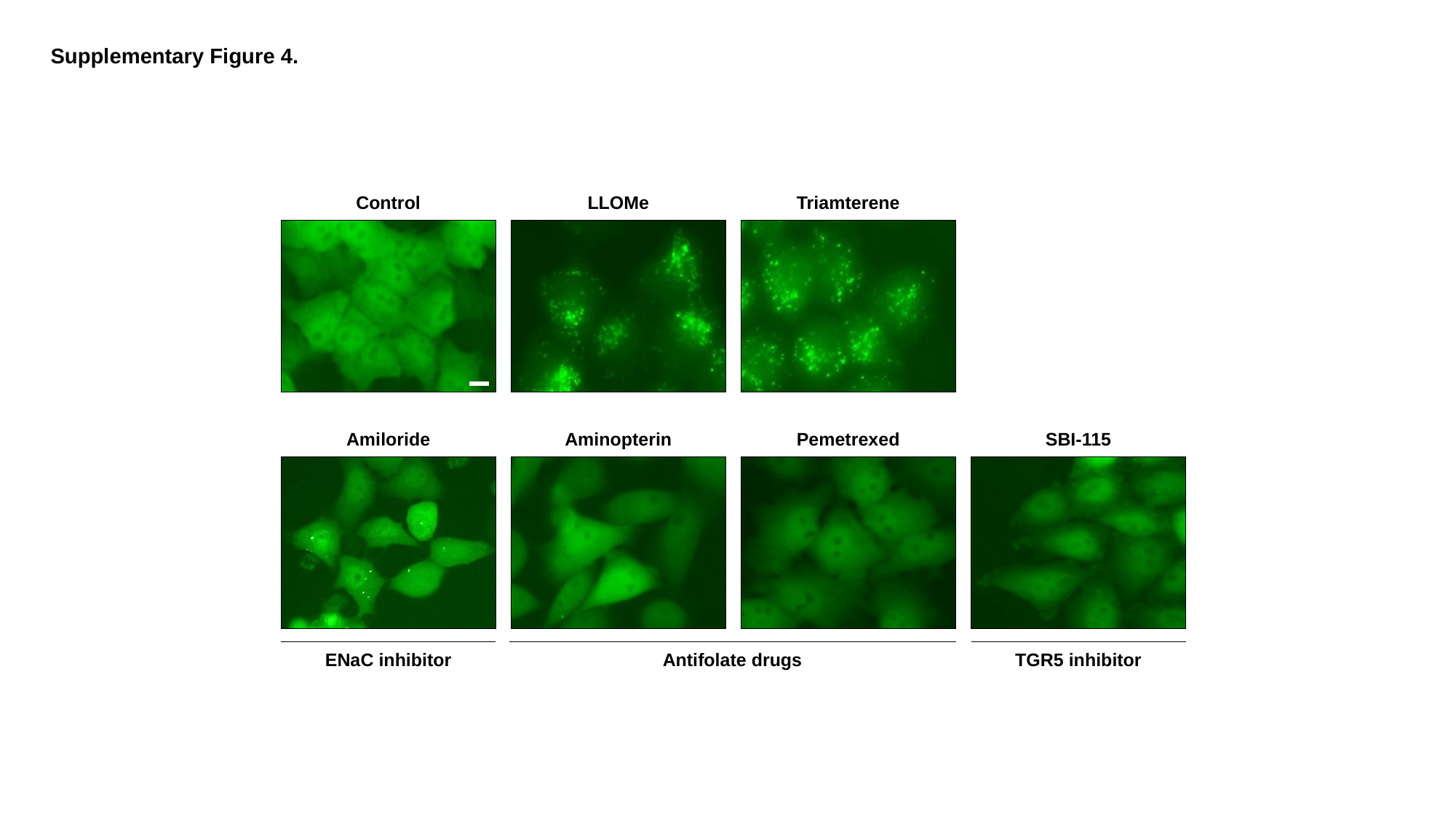

Supplementary Figure 4.
Control
LLOMe
Triamterene
Amiloride
Aminopterin
Pemetrexed
SBI-115
ENaC inhibitor
Antifolate drugs
TGR5 inhibitor
