# Supplementary material for: Triamterene induces autophagic degradation of lysosome by exacerbating lysosomal integrity
Source: Arch Pharm Res. 2021 Jun 7;44(6):621–31. doi: 10.1007/s12272-021-01335-5 (PMC8254722; doi:10.1007/s12272-021-01335-5)
Supplement: Supplementary file 2 — Electronic supplementary material 2 (DOCX 13 kb) [file 12272_2021_1335_MOESM2_ESM.docx]

**Supplementary Figure 1. List of lysophagy regulator.**

List of putative lysophagy regulators from a metabolite library screening.

**Supplementary Figure 2. Efficiency of ATG5 and SQSTM1 siRNA.**

(A and B) HepG2 cells transfected with siRNA against ATG5 or SQSTM1 were harvested and analyzed by western blotting with ATG5 antibody (A) and SQSTM1 antibody (B).

**Supplementary Figure 3. Effect triamterene on organelles.** Both HeLa cells (WT) and ATG5-deficient HeLa (ATG5 KO) cells were treated with triamterene for 2 hours. The cells were harvested and analyzed by western blotting with antibodies for indicated organelles marker protein.

**Supplementary Figure 4. Effect of various chemical on distribution of GFP-Gal3**

HepG2/GFP-Gal3 cells were treated with indicated drugs [LLOMe 750 µM, triamterene 200 µM for 2 hours and ENaC inhibitor (Amiloride 5 mM), antifolate agents (Aminopterin 600 µM, Pemetrexed 600 µM), and TGR5 inhibitor (SBI-115 200 µM)] for 48 hours. Then the cells were imaged with GFP-Gal3. Scale bar: 10 µm.
